# Neuroinflammatory *In Vitro* Cell Culture Models and the Potential Applications for Neurological Disorders

**DOI:** 10.3389/fphar.2021.671734

**Published:** 2021-04-23

**Authors:** Ye Peng, Shifeng Chu, Yantao Yang, Zhao Zhang, Zongran Pang, Naihong Chen

**Affiliations:** ^1^ School of Pharmacy, Minzu University of China, Beijing, China; ^2^ State Key Laboratory of Bioactive Substances and Functions of Natural Medicines, Institute of Materia Medica and Neuroscience Center, Chinese Academy of Medical Sciences and Peking Union Medical College, Beijing, China; ^3^ College of Pharmacy, Hunan University of Chinese Medicine, Changsha, China

**Keywords:** neuroinflammation, blood brain barrier, glial cell, co-culture, neurological disorders

## Abstract

Cell cultures are used in pharmaceutical, medical and biological sciences. Due to the ethical and cost limitations of *in vivo* models, the replaceable cell model that is more closely related to the characteristics of organisms, which has broad prospects and can be used for high-throughput drug screening is urgent. Neuronal and glial cell models have been widely used in the researches of neurological disorders. And the current researches on neuroinflammation contributes to blood-brain barrier (BBB) damage. In this review, we describe the features of healthy and inflamed BBB and summarize the main immortalized cell lines of the central nervous system (PC12, SH-SY5Y, BV2, HA, and HBMEC et al.) and their use in the anti-inflammatory potential of neurological disorders. Especially, different co-culture models of neuroinflammatory, in association with immune cells in both 2D and 3D models are discussed in this review. In summary, 2D co-culture is easily practicable and economical but cannot fully reproduce the microenvironment *in vivo*. While 3D models called organs-on-chips or biochips are the most recent and very promising approach, which made possible by bioengineering and biotechnological improvements and more accurately mimic the BBB microenvironment.

## Introduction

The *in vivo* experiment is an essential step in drug development, which helps to detect the drug efficacy, the development value, and the targeted therapeutic agents in the treatment of diseases. However, in the studies of neurological disorders, *in vivo* models, such as the APP/PS1 transgenic AD model, experimental ischemic stroke model [middle cerebral artery occlusion (MCAO)] or MPTP effect in the treatment of PD model and smoothly into clinical trials of new chemical entities (NCEs), are few successful. This may due to the differences in biology, complex brain structure, metabolism, and signal transduction cascade reaction of pathogenesis between the human body and experimental animals. In 3- dimensions (3D) cell culture.

In the past few decades, the neuron and glial cell models provided many valuable research tools for neurological disorders. In recent years, neuroinflammation is a hot spot in the research of neurological disorders, which also makes people focus on the activation and inflammatory response of astrocytes and microglia. The double-edged sword role of glia and its driving role in neurodegenerative diseases has been evoked an intense debate. Compared with other central nervous systems (CNS) resident cells, glial cells have the largest number and the widest distribution, in that some of them participate in innate immunity. They help monitor the physiological environment and act as the first line of defense when the central nervous system is under attack. Glial cells play different roles in different backgrounds of neuroinflammatory diseases. Different glial cells coordinate and interact with neurons and other brain cells to maintain homeostasis and shape a unique immune response to the CNS. To understand the pathophysiological mechanisms underlying neurodegenerative disease and establish a high throughput screening (HTS) model of drugs for nervous system diseases, it is imminent to have appropriate models for *in vitro* studies with neuroinflammation.

Clinical and experimental researches have shown that blood-brain-barrier (BBB) dysfunction is an early marker of a variety of neurological disorders, including inflammatory autoimmunity, traumatic diseases, neurodegenerative diseases, epilepsy, and stroke. BBB is actively involved in inflammatory events, and various inflammatory stimuli have been shown to affect BBB function in several aspects (Sonar and Lal, 2018). Secondly, due to the existence of the BBB, the CNS has an immune advantage. The traditional view is that neuroimmunology is a protective response mechanism to the brain, but excessive neuroinflammation during the development of the disease may lead to harmful effects, in which the change of BBB permeability is the first step of the occurrence of the disease (Hendriksen et al., 2017). The neuroinflammatory response is characterized by activation of microglia and astrocytes, infiltration of peripheral white blood cells, and release of pro-inflammatory mediators, which exacerbate the destruction of the blood-brain barrier, further amplifying the inflammatory response and aggravating neuronal damage, thus forming a vicious cycle (Zhu et al., 2018). Significant advances have been made in the regulation of BBB function in homeostasis and neuroinflammation, in that this review focuses on the cellular model that simulates BBB in neurological disorders.

BBB is an essential immune part of the CNS, it composites of different types of endothelial cells and immune cells, and the simplex cell is inappropriate to uncover the complex system, so scientists invented cell co-cultured. Traditional cell culture methods usually exist in 2-dimensional (2D) form, with cells laid flat on the surface of the plastic culture, making it easy to grow, but lacking the role of extracellular matrix (ECM), all cells are free to contact with nutrients. In 3- dimensions (3D) cell culture, cells grow in the ECM that they produce, so the nutrients and oxygen they are exposed to are more similar to the environment *in vivo*. When they grow in 3D, the cell morphology and growth characteristics are more consistent with normal brains. The generated ECM can improve the signal transduction between cells and form an interaction network between cells more similar to the *in vivo* environment (Korhonen et al., 2018). Although modeling in 3D culture can better simulate the environment *in vivo* and improve the replication of the model of neurological disease *in vitro*, 3D modeling also has many challenges. For example, in the nervous system, cells in different brain regions may contain the same cell types, but different proportions of cells may perform completely different functions, and 3D modeling still cannot solve the problem of cell proportions. Another example is that most of the existing detection equipment and technology are supporting 2D models, which cannot be directly applied to 3D models. The superiority of the 3D cell model has been gradually recognized, it will be the focus of the CNS disease and drug research field. At present, the *in vitro* 2D cell model is still used widely in most drug development. With the progress of technology, the use of a 3D model to rebuild the body cells *in vitro* microenvironment application is an irresistible trend.

Improved *in vitro* models allows the study of molecular mechanisms to be carried out in a simple and reproducible manner. This review provides an overview of the wide application of cell culture in many different fields, the 2D and 3D neuroinflammatory cell models, and their potential applications in nervous system diseases, which have great application value in the high-throughput screening of drugs for the nervous system diseases.

## Features of Healthy and Inflamed Blood-Brain Barrier

In both health and disease, the BBB acts as a mediator between the periphery and the CNS (Daneman and Prat, 2015). Peripheral immune cells pass through the BBB to enter the brain parenchyma to clear microorganisms or cell debris, and abnormal cell transport functions of the cerebrovascular system may lead to the development or aggravation of neurological disorders. The cells that make up the BBB can communicate strongly with each other, which include endothelial cells, pericytes, astrocyte podocytes connecting the endothelium and pericytes, and supplementary cells such as microglia, neurons, interneurons, white blood cells, and oligodendrocytes (Jackson et al., 2019). Under physiological conditions, the BBB has a three-layer defense system. The first layer is endothelial cells, forming the blood vessels to provide scaffolds for connecting adhesion molecules to ECM (Ayloo and Gu, 2019). The last defense line is astrocytes, especially the extended end-feet surrounding ECM and pericytes, against harmful proteins and molecules. They regulate brain blood flow, neuron function, and the formation of tight connections (Langen et al., 2019). Pericytes are inserted in the basement membrane of capillary endothelial cells between astrocytes and ECM, form the scaffold of endothelial cells, and sends paracrine messages through direct contact with endothelial cells (Edwards and Bix, 2019).

CNS ischemia or mechanical damage, such as spinal cord injury (SCI), traumatic brain injury (TBI), or ischemic stroke, and is often accompanied by inflammation, and chronic neuroinflammation also occurs in neurodegenerative diseases. Under continuous inflammatory stimulation or inherent diseases (as aging and genetic defects), CNS cannot maintain a steady-state, which exacerbates chronic neuroinflammation. In the process of inflammation and disease progression, reactive glial cells secrete a large number of pro-inflammatory mediators and participate in the opening of the BBB, leading to changes in the permeability of the BBB and infiltration of peripheral blood immune cells (Yang and Zhou, 2019). Deconstruction of pericytes and glial cells around capillary endothelium affects vascular permeability and is involved in neuroinflammation. The capillary BBB and the outer compartment, pericytes, and astrocytes coexist to maintain the functional characteristics of the BBB. This unique anatomical structure allows pericytes to communicate with endothelial cells and glial cells including astrocytes and microglia (Giannoni et al., 2018). *In vivo* studies have shown that pericytes modification and response are closely associated with inflammatory changes in CNS lesions, including ischemic stroke (Fernández-Klett et al., 2013), Alzheimer’s disease (Giannoni et al., 2016; Love and Miners, 2016), epilepticus (Milesi et al., 2014; Klement et al., 2019), TBI (Choi et al., 2016), and peripheral CNS diseases such as SCI (Göritz et al., 2011).

Cerebral edema accompanied by neuroinflammatory lesions is also the most typical lesion of CNS inflammation. In the case of ischemic stroke, inflammation first damages the tight connections of the brain, leading to the free movement of molecules in the endothelial cells (Krueger et al., 2015) and a loss of endothelial integrity (Krueger et al., 2013). The role of endothelial cells in BBB permeability 24 h after stroke has been demonstrated. Subsequently, astrocyte swelling and pericyte contraction, and capillary diameter reduction occur. This acute mechanism after ischemia leads to astrocyte death, exacerbation of cytotoxic edema, and BBB dysfunction (Edwards and Bix, 2019).

In the modeling of the BBB, endothelial cells, pericytes and astrocytes are often used to simulate a complete barrier. Considering the number of all these cells and the related proteins they express, as well as the interactions between cells, creating an optimal static or dynamic BBB model system is challenging (Jackson et al., 2019).

## Single-Cell Model of Neurological Disorders

Over the past few decades, BBB models have been constructed *in vitro* using primary and immortalized cell lines from several species, including mice, rats, pigs, cattle, and humans. Although the primary cells have significant physiological relevance to the body, the separation is laborious and time-consuming. And for basic scientists without fixed clinical collaborators, the sources of healthy human tissues are very limited. Besides, the primary cells of other species also have the problem of interspecific differences. Therefore, commercial immortals, which are simple and easily purchased, occupy a certain position in the research field, such as cell lines simulating neurons: rodents (PC12), humans (SH-SY5Y), rodent endothelial cell lines (bEnd.3 and TR-BBB), human brain endothelial cell line (HBMEC), BV2 microglia cell line and HA astrocytes cell line. In recent years, human-derived induced pluripotent stem cells (iPSC) can differentiate into endothelial cells, glial cells, and other different cells to simulate *in vitro* BBB modeling (Jackson et al., 2019). Cell culture technology has increased the reproducibility of research on diseases of the nervous system, cell experiment before clinical trials to minimize the body is the purpose of the experiment, has become an important tool of pharmacology and toxicology study.

### Main Intestinal Cell Lines

#### PC12 Cell Lines

PC12 belongs to the adrenal phaeochromocytoma cell line, was isolated from an adrenal medulla tumor (Greene and Tischler, 1976). Like other adrenal pheochromocytes, PC12 cells synthesize and store dopamine (DA) and sometimes norepinephrine (NA) (Westerink and Ewing, 2008). Nerve growth factor (NGF) can induce PC12 cells to differentiate into a morphology similar to primary sympathetic neurons, and its phenotype is similar to that of mature sympathetic neurons (Westerink and Ewing, 2008). The PC12 cell line is widely used for neurobiological and neurochemical research, including studies on neurotoxicity (Tang et al., 2019), neuroprotection (Lee et al., 2018), neurosecretion (Barlow et al., 2018), neuroinflammation (Yin Z. et al., 2020), synaptogenesis (Wiatrak et al., 2020), and as a principal dopaminergic model in molecular neuroscience (Vaudry et al., 2002). In addition to studying various aspects of neuronal phenotypes as differentiated neuron-like cells, PC12 is also an excellent *in vitro* tool for studying the pathogenesis and progression of various neurological disorders, such as glutamate excitotoxicity (Kritis et al., 2015), Parkinson disease (PD) (Loeffler et al., 2016), AD (Ştefănescu et al., 2019), epilepsy (Thiel et al., 2015), and ischemia (Lahiani et al., 2018). However, because it is a rat-derived cell line and needs to be induced by neurotrophic factors to differentiate into a neuron-like morphology, it is different from the neurons in the body, so it often compares with other cell lines when selecting it as a research object (For example, SH-SY5Y (Wan et al., 2019)) or the primary neuron together corroborated.

#### SH-SY5Y Cell Lines

Neuroblastoma SH-SY5Y cell line is an immortal cell line widely used in *in vitro* models of neurological disorders. The subline of the SK-N-SH cell line, which was established from a 4-year-old female bone marrow aspiration biopsy (Biedler et al., 1978). SH-SY5Y has been widely used as a research object of PD because it has a mechanism to synthesize DA (activate dopamine-β-hydroxylase) and NA (Xicoy et al., 2017). But not limited to this, it is also widely used in other areas of neurological disorders, including AD (de Medeiros et al., 2019), neurotoxicity (Wang et al., 2019), glutamate excitotoxicity (Vucicevic et al., 2020), ischemia (Cui et al., 2020) or amyotrophic lateral sclerosis (ALS) (Giannini et al., 2020) and other diseases. However, SH-SY5Y retains the characteristics of cancer including the instability of the proliferation, differentiation, and metabolism because it is isolated from neuroblastoma derivatives. Therefore, it is necessary to specify the source of the cells, in that the differences between cells may be due to epigenetic characteristics that can explain these differences, to select a suitable *in vitro* experimental model (Xicoy et al., 2017).

#### N2a Cell Lines

The Neuro-2a (N2a) cell line, derived from a mouse brain neuroblastoma, can be differentiated into neuronal-like cells (Xicoy et al., 2017) which has been largely employed and a well-characterized system to study signal pathways (Chen et al., 2017), secretory events (Gutiérrez-Martín et al., 2011) and neuronal differentiation (Huo et al., 2020). N2a cells increased expression of amyloid precursor protein (APP) and functional ionotropic P2X and metabotropic P2Y receptors (Miras-Portugal et al., 2015). P2X7 receptors are related targets of the P2 receptor family and are abundantly expressed in microglia, astrocytes, and neurons (Sperlágh and Illes, 2014). The other is the P2Y2 receptor, which is mainly involved in the processing and regulation of APP by activating non-amyloidogenic pathways (Erb et al., 2015). As thus, N2a cells are widely used in degenerative diseases of the CNS.

#### Microglia

Microglia originate from erythromyeloid progenitor cells in the embryonic yolk sac and migrate into the brain to propagate, spread, and ramify (Hammond et al., 2018). Their homeostasis is affected by several factors including transforming growth factor-β (TGF-β) and colony-stimulating factor 1 receptor (CSF1R) (Sarlus and Heneka, 2017). The activation of matrix metalloproteinase (MMPs) may cause BBB injury. In the LPS-induced microglia inflammation model, inhibiting MMP3 and MMP9 can attenuate the expression of pro-inflammatory cytokines and inducible nitric oxide synthase (iNOS) secreted by microglia (Niranjan, 2018). It can also regulate the release of chemokines by up-regulating the expression of MMP-3 (Connolly et al., 2016). The current research on microglia is mostly focused on discussing its double-edged sword function in neurological disorders (such as AD (Sarlus and Heneka, 2017)). The classical theory believes that under appropriate stimulation, classically activated pro-inflammatory cell phenotype (M1) polarization plays a role as the first line of defense in innate immunity, but it usually occurs in the first few hours or days. Plasma cells exist in various activated states in injured tissues. After they are exposed to pro-inflammatory cytokines interferon-γ (IFN-γ), tumor necrosis factor-α (TNF-α), and cell or bacterial fragments, they will polarize to the M1 phenotype. These cells then produce pro-inflammatory cytokines, chemokines, redox molecule (NADPH) oxidase, and express high levels of iNOS to produce NO. Microglia will exhibit M1 responses when exposed to LPS or IFN-γ in many experimental models (Martinez and Gordon, 2014). A similar but sterile M1 response usually occurs in the absence of microorganisms, due to TBI, ischemia, or chemical exposure (Lan et al., 2017; Sen et al., 2020). M2 type macrophages can promote inflammation subsidence through anti-inflammatory factors, thereby inactivating the pro-inflammatory cell phenotype and rebuilding homeostasis. The M2 polarization of microglia is similar to that of peripheral macrophages (Fenn et al., 2012; Ting et al., 2020), producing different IL-4 and IL-mRNA profiles. However, some studies have shown that activation pathways outside the standard M1/M2 polarization paradigm are getting more and more attention, instead of using any typical markers of specific activation states alone. It is becoming increasingly clear that any research that attempts to determine whether microglia are present in different activation or polarization states cannot rely on just one or two markers but needs to examine multiple markers and evaluate morphological tables. Understanding the dynamic regulation function of microglia better will by distinguishing the status of resident and infiltrating macrophages, metabolism, mitochondrial function, etc. (Orihuela et al., 2016). Our views on microglia are changing with new technologies and research findings. The concept of M1/M2 is mainly applicable to the inflammation when macrophages are derived from infiltrating monocytes in diseased tissue reaction. The morphology of microglia in the non-diseased brain showed fine, radially directed branches to a small ellipsoid. In diseased tissues, microglia behave completely differently, the cell volume increases, shortens, and the protrusions decrease, sometimes showing a clear phagocytic appearance, which form is designated as the activated state. In the contrast, microglia are designated as the resting state under physiological conditions. Nimmerjahn et al. (Nimmerjahn et al., 2005) explained the limitations of this M1/M2 framework to microglia came from two-photon imaging, they found that microglia are very active in a presumed resting state and constantly observe their microenvironment. Besides, the destruction of the BBB triggers immediate and local activation of microglia, transforming their behavior from patrolling to protecting the injured site. Using flow cytometry and deep RNA sequencing technology to analyze spinal microglia the activation situation was studied. The result showed that analysis of microglia in resting-state found 29 genes that distinguish them from other CNS cells and peripheral macrophages/monocytes (Chiu et al., 2013). Single-cell sequencing also revealed significant differences in expression profiles, function, survival, and ultrastructural characteristics between microglia and monocyte-derived macrophages (Yamasaki et al., 2014; Kim et al., 2016). Up to now, the polarization mode of microglia is still under discussion and debate. The function of microglia has been a research hotspot in neurobiology for a long time. Researchers have developed and used a variety of microglia cell lines to study the function of microglia *in vitro*. Widely used are the neonatal microglia (P1), primary cultured neonatal microglia (P1-P2), microglia cell lines (N9 and BV2), embryonic stem cell microglia (ESDMS) 24, and RAW264.7 macrophages. However, the use of culturing primary microglia is limited with the few numbers of cells obtained and the technology is time-consuming. The two commonly used cell lines produced by the replacement are BV2 and N9 microglia from rats and mice to quickly produce large numbers of cells, respectively.

BV2 cell lines: Blasi’s research group adapted the successfully immortalizing murine macrophages (Blasi et al., 1985) to form the BV2 microglial cells (Blasi et al., 1990). The BV2 cells were expressed 90% positive for microglia cell markers, containing macrophage-1 (MAC-1) and MAC-2, but negative for MAC-3 antigens (Horvath et al., 2008).

N9 cell lines: N9 microglia cells are derived from the mouse brain and have many phenotypic characteristics similar to the primary mouse microglial cells. Hickman’s group conducted some experiments on N9 cell lines, and the results showed that N9 cell lines can clear β-amyloid precipitate (Aβ) and express the regulatory receptors of microglia cells. Also, the expression of scavenger receptor A and CD36 was decreased when N9 was incubated with TNF-α, and Aβ absorption was also decreased, consistent with their results obtained from primary mouse microglia (Hickman et al., 2008). Moreover, this cell line has a similar phagocytic function to primary microglia and expresses inflammatory factors (Vaz et al., 2019). However, because they were genetically modified, their proliferative ability and adhesion increased compared with the primary microglia (Stansley et al., 2012). N9 microglia have also been shown to be similar to BV2, including up-regulation of pro-inflammatory genes and expression of iNOS and inflammatory factors (Fleisher-Berkovich et al., 2010).

However, none of these immortalized cells expressed the characteristics of adult microglia. The primary microglial cells isolated and cultured from newborns were closest to adult microglia cells, and the microglia-specific genes were rarely expressed in microglia cell lines (Butovsky et al., 2014).

#### Astrocytes

Overcame many difficulties in purifying astrocytes to investigate astrocytes in the early 1980s, such as cells freshly isolated from the brain that always contained a mixture of Microglia and Progenitor cells (McCarthy and de Vellis, 1980). However, subsequent studies have shown that although the serum and other nutrients in the astrocytes isolated by this method are necessary to maintain their physiological functions, such culture conditions may irreversibly alter the transcriptome of astrocytes (Foo et al., 2011). This may make the isolated astrocytes different from the physiological astrocytes *in vivo* (Allen et al., 2012). These studies suggest that a perfect culture system designed to mimic “normal” astrocytes *in vivo* may require a combination of several characteristics, including specific nutritional support, the right matrix, and possibly other unknowns. Total transcriptome analysis of sera-free 3D cultures of immune cells may provide a more accurate model for understanding the function of astrocytes in normal, healthy brains (Liddelow and Barres, 2017).

Astrocytes have been shown to exist in two different response states. One is neuroinflammation-induced reactive astrocytes, known as type A1, and the other is ischemia-induced reactive astrocytes, known as type A2. This term is similar to the earlier terms for M1 and M2 macrophages, which describe the polarization of microglia. If A1 and A2 are the only two states of astrocytes, then they may exist with a heterogeneous mix of A1 and A2 present in the body. Transcriptome analysis of reactive astrocytes revealed that A1 neuroinflammatory reactive astrocytes upregulated many previously proven synaptic destructive genes such as complement cascade genes, suggesting that A1 may have deleterious functions. Notably, LPS induction failed to induce A1 type response in microglia-deficient mice (CSF1R knockout), suggesting that microglial activation may be a necessary condition for A1 astrocyte production (Liddelow et al., 2017; Hinkle et al., 2019). In contrast, A2 reactive astrocytes upregulate many factors such as thrombopoietin neurotrophic factors, which promote neuronal survival and growth and promote synaptic repair, in that A2 may have beneficial or restorative functions. Consistent with this, ischemia-induced reactive astrocytes have been shown to promote recovery and repair of the CNS (Gao et al., 2005; Hayakawa et al., 2014). However, these phenomena are not enough to explain the function of astrocytes. For example, what are the substances that induce the intercellular interactions of reactive astrocytes, as well as their mechanisms of action and signaling pathways, are not clear, further research and a more comprehensive understanding of the response pathways of astrocytes are needed (Liddelow and Barres, 2017). In the course of conventional neurologic research, two commonly used cell lines are HA and C8-D1A astrocytes cell lines which are derived from humans and mice, respectively. Both of them are widely used in research SCI (Zhang Z. et al., 2018), BBB simulated *in vitro* (Booth and Kim, 2012), and ischemic (Chen et al., 2018).

#### Endothelial Cells

In addition to the above-mentioned neurons and specific immune cells in the brain, another important cell that constitutes the BBB is the endothelial cell. Endothelial cells play a leading role in regulating the brain microenvironment. The endothelium of the brain differs from the endothelium of most other tissues in that the tight connections within the endothelium of the brain are much tighter and more complex. Occludin and Claudins are the transmembrane proteins that make crucial contributions to its tight junction structure. Occludin’s main function is to regulate the tight connections of brain cells. In the mechanism of the BBB, the expression of the proteins Claudin 3, 5, and 12 contributes to the increase of trans-epithelial electrical resistance (TEER). High expression levels of proteins in brain endothelial cells include junction adhesion molecules, linking proteins in the interaction domain of multiple proteins, Calcium-dependent serine protein kinase (CASK), Membrane-associated guanosine kinase with a reverse orientation of the protein interaction domain, and more (Abbott et al., 2006). Transmitters and modulators released by BBB cells allow complex signals to be transmitted between neurovascular unit cells. For example, under normal circumstances, the tight junction of the BBB may open to allow growth factors and antibodies to enter the brain, while inflammation may lead to brain edema. Maintaining endothelial health helps to prevent the development of neurodegeneration. Commonly used brain endothelial cells include b.End3, TR-BBB/TM-BBB, HCMEC/D3, and HBMEC cell lines.

TR-BBB/TM-BBB cell lines: Immortalized brain endothelial cell lines were established from Tg mice (TM-BBB) and Tg rats (TR-BBB). Both of them express endothelial markers and possess Glucose transporter type 1 (GLUT1) and p-glycoprotein, which play a transporter function. The [3H]3-O-Methyl-D-glucose (3-OMG) expression values were very similar to those expressed in endothelial cells *in vivo*. TM-BBB cells exhibited efflux transport activity against cyclosporin A.TR-BBB cells also expressed multidrug resistant-associated protein 1 (MRP1 (ABCC1)) and L-type/large neutral amino acid transporter 1 (LAT1(SLC7A6)) mRNA (Hosoya et al., 2002).

HCMEC/D3 cell lines: A tumor cell line derived from human temporal lobe microvascular and later immortalized during SV40 large T-antigen transduction. The expression of protein transporters and BBB receptors are commonly used to study the inflammation of brain endothelium, the mechanism of Aβ production, and the permeability of BBB. However, compared with the primary cerebral endothelial cells, this cell line had a lower Teer value, and the expression levels of Occluding and Claudin-5 were not consistent. The function of brain endothelial cells cannot be completely replaced (Dubey et al., 2019).

Human Brain Microvascular Endothelial Cell (HBMEC): HBMEC is derived from hiPSCs or the human brain tissue. HBMEC and BBB cells have many physiological similarities. For example, their expression of claudins, occludins, junctional adhesion proteins, and transporters are all related to BBB. hBMECs are used as an effective *in vitro* BBB model for brain research, modeling the homeostasis and the changing of the physiology and characteristics (Dubey et al., 2019).

#### Induced Pluripotent Stem Cells

Induced Pluripotent Stem Cells (iPSCs) are self-renewing cells capable of differentiating into different types of cells, including microglia, astroglia, endothelia, and various neuronal cell lines. iPSCs have broad prospects in human disease modeling *in vitro* and are effective tools for researchers to study the occurrence and development of diseases and the use of drugs (Haston and Finkbeiner, 2016; Poon et al., 2017). iPSCs can proliferate and differentiate into many different neuron subtypes (Sances et al., 2016; Engle et al., 2018; Real et al., 2018; Chang et al., 2020), astrocytes (Barbar et al., 2020), microglia (Abud et al., 2017), oligodendrocytes (Marton et al., 2019), endothelial cells (Paik et al., 2018) and pericyte (Stebbins et al., 2019), which allows the study of individual cells and their functions and interdependence in the brain. iPSC plays an important role in establishing 3D cell culture models *in vitro* (Campisi et al., 2018).

### 2D Co-Culture Model

The original co-culture training means is separate cells culture, but with a cell culture system to stimulate the other cells, to achieve the result of simulation trained, stimulate microglia with neuron culture supernatant, observes the polarization state of the microglia, and vice versa in the cultivation of the microglia supernatant stimulate the nerve cells, observe the activated microglia and the influence of the discharge of neurons. For example, Guo et al. stimulated SH-SY5Y cells by the BV2 culture supernatant, observed microglia activation, and inflammatory cytokines secretion, which can cause apoptosis of neurons and synapses damage (Guo et al., 2019). A more straightforward co-culture model involves growing both cells in the same culture system to mimic the environment in the brain. For example, Shi et al. isolated primary glial cells and neurons from mice for glia-neuron co-culture. They directly plated primary neurons expressing P301S tau on top of the glia (Shi et al., 2017). Simple cell co-culture is only carried out on the rigid plastic surface, and the rigidity of plastic Petri dishes is not consistent with physiology. However, due to its low cost, simple technical method, and high instrument matching degree, it is still a very useful model in the evaluation of drug cytotoxicity, but it cannot adapt to more complex research problems. To improve on simple cell models, the researchers developed 2D co-culture cell models as well. The integrity of the BBB cell model *in vitro* was enhanced by co-culture of neurons, glial cells, endothelial cells, and/or pericytes. For example, wells filled with semi-permeable membranes are used to assist cell modeling. These cross-holes allow the implantation of brain-derived cells, simulating the closeness and growth distance of cells in the luminal and outside the luminal surfaces of BBB. The most widely used is the Transwell model. Transwell membranes are usually made of polycarbonate or polyethylene terephthalate with pore sizes ranging from 0.4 to 3.0 μm. Cells are seeded in the chamber and on the surface of the chamber and then a series of downstream analyses can be performed (such as TEER). TEER measurements help to assess the integrity of the BBB (Wimmer et al., 2019). Transwell is widely used because it is easier to use, faster to install, and less expensive. They have standardized operating instructions and readout methods and are capable of directly testing 96-well plates. Though transwell can contain a variety of cells to simulate the interaction between cells, it still has some limitations, such as the lack of dynamic interactions between cells. Tanswell celling only allows cell growth over time, at the same time can provide closely observe a functional difference, and there is no fluid flow or cell rearrangement of the assessment (Jackson et al., 2019). The static transwell model lacks fluid shear stress, while the conventional dynamic *in vitro* BBB model lacks fluid shear stress and can only be simulated in single-cell conditions. To solve the limitation of these two aspects, the researchers have developed a microfluidic blood-brain barrier (mBBB), which can achieve in a dynamic environment to cultivate and can realize cultivate membrane to separate cells, similar to that of the transwell. As early as 2012, Booth and Kim use b.End.3 and C8-D1A jointly developed a microfluidic μBBB chip, which in the dynamic environment and the cultivation of the relatively thin film (10 microns) to simulate BBB (Booth and Kim, 2012). This provides a reference and foundation for the development of a Microfluidic system in the future. Although 2D cell co-culture has many shortcomings, 2D cell culture can still be used for preliminary screening of drug BBB permeability and the study of BBB integrity after systematic treatment. No matter in direct or indirect co-culture mode or BBB simulation by transwell, the results need to be confirmed by a more complex dynamic model.

### 3D Co-Culture Model

Cell culture is often restricted by detaching from the ECM when simulating the cell environment in the body. More and more evidence prove that the 3D culture system represents a more accurate physiological environment, which is different from the traditional cell culture method. Compared with the 2D co-cultivation method, the 3D can better simulate the way of accurate physiological environment. Among the cell models that simulate the CNS, the BBB model is the most widely studied.

### 3D Model of the BBB

Co-culture of cells in ECM protein solutions helps to assess the complexity of BBB structure in 3D modeling. ECM provides soft reticular support for neurons, glia, endothelial cells, and pericytes to adapt to their growth, migration, and expansion (Jackson et al., 2019). Besides, ECM also provides a place for interaction between BBB cells. Culturing and observing cell interaction in a 3D matrix help to simulate the close relationship between cells. The close association between cells can be observed in real-time under an electron or optical microscope, or after fixation. Bang et al. developed a 3D BBB platform that separates the cell types in a single device by independently providing different types of media (Bang et al., 2017). One vascular channel (VC) connects to the lumen of the vascular network, and the other neural channel (NC) provides mediators for nerve cells. This allows astrocytes to be in direct contact with endothelial cells while being spatially separated and simulates BBB’s perivascular network morphology and synaptic structural characteristics, which is useful in screening for brain-targeted drugs in neurodegenerative diseases. However, a major limitation of the earliest 3D modeling systems was the inability to determine the functional integrity of the cell or BBB using TEER or dye permeability methods. Furthermore, despite structural integrity, the model is generally unable to assess the perfusion mechanics between endothelium-pericytes suspended in an ECM scaffold (Jackson et al., 2019). Previous studies reported *in vitro* neurovascular unit (NVU) models to investigate BBB functions using Transwell or microfluidic devices. Uwamori et al. introduced a 3D culture model on a microfluidic platform that allows the 3D culture of human neural stem cells (NSC), HBMEC, and human mesenchymal stem cells (MSC) for the construction of neurovascular tissue (Uwamori et al., 2017). They constructed in neurons and capillary neural vascular tissue, and then build the structure of 3D NVU: cell culture in the fibrin, matrix, and hyaluronic acid composition of ECM in the hydrogel, and on microfluidic platform induction of neurogenesis and angiogenesis in the process of cultivating the NSC and BMEC, compared with the traditional configuration (such as Transwell systems) microfluidic system has obvious advantages, can be used to study via the interaction between brain cells and brain function research, but also can be used as a treatment strategy of a drug screening study. These microfluidic devices dynamically assess BBB integrity, permeability, and cell invasiveness by real-time measurement of TEER, dye permeability, and fluorescent cell imaging (Sivandzade and Cucullo, 2018). When iPSC-derived neurons, astrocytes, endothelial cells, and pericytes are co-cultured in these micro-controlled flow devices, the system can achieve model parameters very similar to the BBB in the physiological environment, and by changing the physiology or disease state of the cell, the system can be quickly applied to BBB modeling in the disease state. Although dynamic microfluidic devices are closer to simulating the physiology of healthy and diseased BBB, these systems are more expensive and require specialized equipment and technical expert guidance to be established, and because the method is relatively new, supporting standardized operations and detection parameters also limit its widespread use (Jackson et al., 2019). In recent years, 3D printing has emerged as an alternative manufacturing method that allows faster, cheaper manufacturing, precise size control, built-in measurement technology, and greater design flexibility (Li et al., 2020). Combining 3D printing technology with the advantages of high throughput detection, automatic detection, and low operating cost of microfluidics solves the problem of the complex multi-step structure of the microfluidic device and can be used as an alternative method of traditional microfluidics manufacturing. 3D printing, as digital manufacturing technology, is the process of adding materials to create objects layer by layer from 3D model data, allowing complex objects to be accurately constructed directly from computer-aided design (CAD) software (Tack et al., 2016). Most 3D printing processes have a similar basic process, in short, the use of CAD software in the form of 3D a digital rendering of the target product, then convert the 3D design to describe the surface stereolithography (SLA) 3D model file format (STL), then, will be cut into 2D layer data further build files, and sent to the 3D printer. The raw materials processed into filaments, granules, or adhesive solutions are automatically added and cured in a layer-by-layer fashion to produce the desired product (Sivandzade and Cucullo, 2018).

### Commonly Used 3D Cell Culture Biomaterials

Due to the rapid development of material chemistry, use scaffolding or solid platform to simulate microenvironment support cell growth has been widely used in copying the ECM model, these materials including metals, plastics, and polymers, such as electrospinning scaffold, by random distribution or arrangement of the electrostatic spinning of nanofibers polyphenylsulfone (PPSu) stents, used for evaluation of neural activity 3D cultivation environment. The results showed that nanofibers successfully supported the adhesion and growth of NSCs and enhanced the differentiation of neurons compared with 2D substrates (Hajiali et al., 2018). Hydrogels are networks of polymers or proteins that can be cross-linked to form a coating on the surface of cells *in vitro*. They can be either natural or synthetic and are used to promote cell attachment and improve their survival. The natural extracellular matrix proteins of cells provide a natural source of hydrogels, such as laminin and collagen. Type I collagen with hyaluronic acid and alginate with laminin are used as cellular scaffolds (Her et al., 2013). Star-PEG-heparin hydrogel has been used to study how the NSC in AD lose regeneration, studies have shown that by using Star-PEG-heparin hydrogels to set up a 3D cell guiding neural microenvironment of reduction, to promote the primary and the induction of NSCs proliferation and nerve generation capability, to study the NSCs how plasticity loss in the AD (Papadimitriou et al., 2018). The tissue-engineered structure of synthetic hydrogels is usually made of a soft and porous matrix to allow oxygen and nutrients to pass through sufficiently to supply cells, such as polycaprolactone (Daud et al., 2012), polyethylene glycol (Schwartz et al., 2015), and even polystyrene, which has been used as scaffolds, cell adhesion, and support. Beduer et al. have also developed a compressible scaffold. Using a hydrogel consisting of sodium alginate and carboxymethyl cellulose (CMC) in combination with simple one-pot cryogenic synthesis, they prepared a cryogen system that assists in the development of extended neural networks from primary cells and can be used for minimally invasive delivery within brain tissue (Béduer et al., 2015).

With the development of biomaterial scaffolds, 3D culture and *in vivo* cell behavior have been realized in scaffolder-free self-assembled aggregated cultures (Dingle et al., 2015), including spheroids, embryos, nerve spheres, and microtissues. Cells can be formed by suspension culture in concave micropores or sponge gel matrix, successfully realizing the communication between various cell types of neural spheres. Biomaterials can also be used to study cell behavior. For example, neurons have also been grown and studied on semiconductor indium phosphate nanowire arrays, demonstrating that the neurites of multiple neurons, guided by InP nanowire scaffolds, exhibit synchronous calcium activity, meaning that cell-to-cell communication via synaptic connections can be identified in this model (Gautam et al., 2017). Neurons grown on nanotubes have shown the advantages of being easy to grow and emitting signals that are highly synchronized with those in the body (Bosi et al., 2015). Miao et al. used the chemical vapor deposition method, through the graphene bubble hole (GCNT web) won the full 3D interconnected carbon nano pipe network, and through training and fluorescent marker a different color of glioma and healthy cortex cells, acquired a new *in vitro* model to study malignant glioma invasion, the model is the study of 3D *in vitro* biological process ideal tool (Xiao et al., 2018). However, the limitations of the scaffold-free 3D culture of nerve spheres and other scaffolds have led to problems such as large size, malnutrition of the culture, and diffusion of oxygen to indoor cells (Cembran et al., 2020).

## Conclusion and Perspectives

**FIGURE 1:**
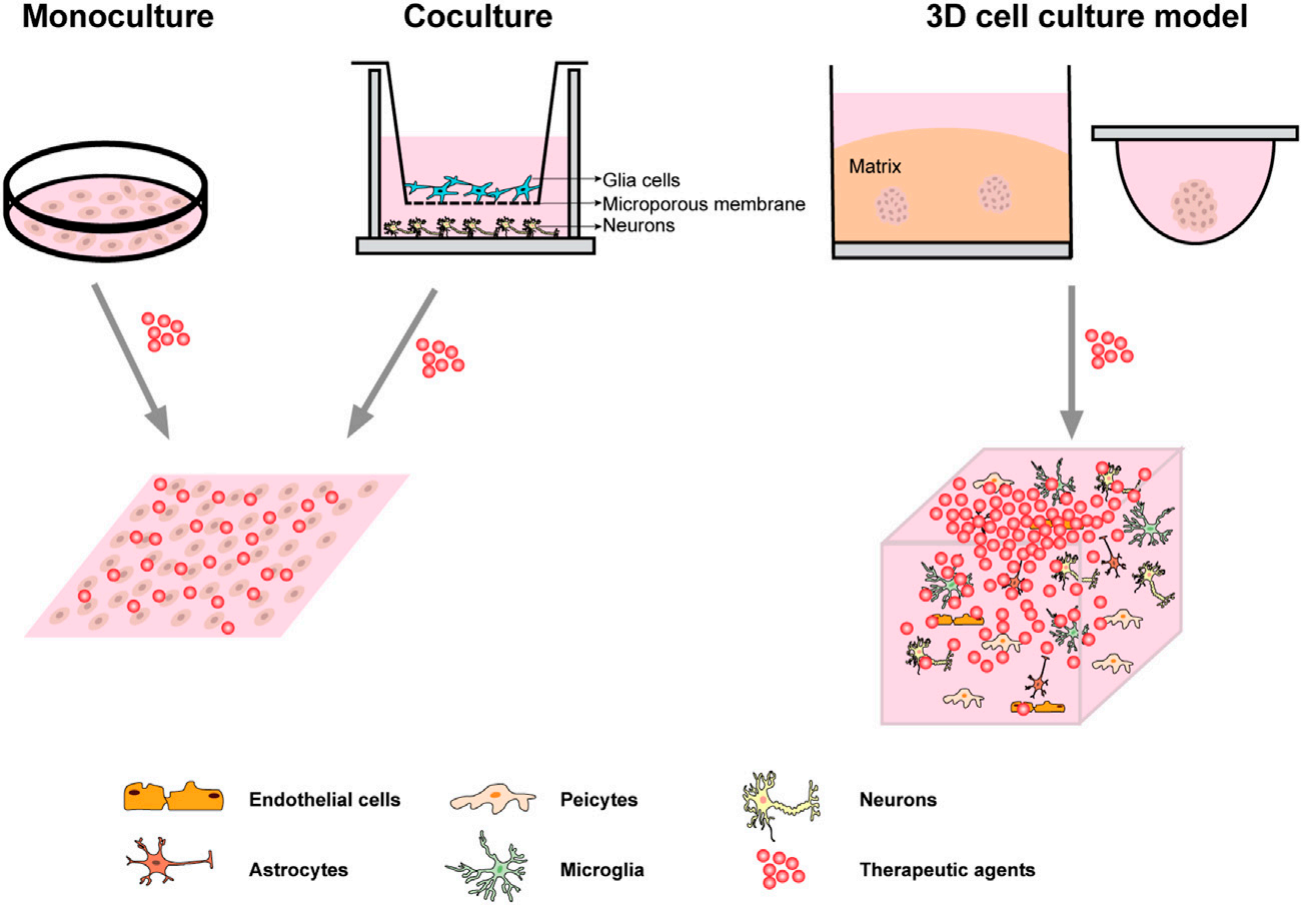
Common cell culture system. Monoculture is usually carried out directly on the rigid plastic culture substrate; A common use of co-culture is to grow both types of cells in the same extracellular fluid using Transwell chambers; The first two systems of culture were homogenous drug distribution when it comes to the treatment medium or the stimulation. The most commonly used 3D culture system is ECM embedded culture and static suspension culture. The neuro-spheroids or microscopic tissue model formed by 3D culture technology can better simulate the microenvironment *in vivo*, and the distribution gradient of drugs *in vivo* can be simulated more accurately when the treatment medium or stimulation is given.

Functional *in vitro* models of the neuroinflammatory have an essential role in the assessment of the risks or benefits of new treatment methods as an alternative to animal studies. Cell lines are cell cultures that develop from a single cell, so that daughter cells are phenotypically and genetically similar to parent cells until or unless they are inclined to differentiate by differentiating agents. They replace primary cells, are crucial in the study of the physiology, mechanisms, and processes of diseases or disorders, and are widely used in drug-screening processes. *In vitro* cell models offer a promising tool for exploring complex human organs such as the brain. It provides supporting data for the researchers' hypothesis and improves the translational value of the *in vivo* model. Typically, this method is practiced in cultures of many cells. Various studies have shown that compared to other models of nerve cells, 3D cell cultures can generalize exactly what the brain's environment looks like and can successfully reconstruct the ECM. As it helps to estimate drug efficacy more appropriately and provides precise directions for researchers to develop promising drug therapies. From simple intracerebral environment monoculture, through co-culture to 3D culture in ECM (Figure 1), *in vitro* model of the nervous system as a tool to create more suitable physiological conditions is of great significance. The selection of the correct research object and preparation methods are essential to maintain the high degree of matching and stability of the cell model, which helps to more accurately simulate the *in vivo* environment and understand the mechanism of the disease state. Future research should focus on producing more accurate *in vitro* models that could be used to develop novel drugs that target the BBB in the CNS, which could provide enormous services to humans (The common brain inflammation 2D and 3D co-culture models are summarized in Table 1).

**TABLE 1:** Brain inflammation 2D and 3D co-culture models.

Model	Cell type(s) & source	Objectives	References
BV2/SH-SY5Y BV2/N2A	Mouse microglia cell lines/Human neuroblastoma cell lines/Mouse neuroblastoma cell lines	Simulation the proinflammatory status of microglia	Liu et al. (2019b)
b.End3/C8-D1A	Mouse brain microvascular endothelial/Mouse brain astrocyte	A dual-layer BBB was formed in the porous model of flowing cell suspension	Booth and Kim, (2012)
Neurons/Microglia	Primary cortical neurons/Mouse brain mixed glial cell	Stimulation the mimic chronic neuroinflammation conditions	Tu et al. (2019); Yin et al. (2020a)
iPSC-PFP-pericytes/iPSC-GFP-BMECS	BC1 and BC1-GFP hiPSC lines	The effects of *in vitro* platforms with varied spatial orientations and levels of cell-cell contact	Jamieson et al. (2019)
HT22/U251	Hippocampal neuronal HT22/Astrocytoma U251 cells	The effects of inflammation, oxidative Stress, and neuron death	Liu et al. (2019a)
Astrocyte/CD3+ T lymphocyte	Primary human fetal astrocyte cultures/Human blood of healthy donors	Stimulation of the changes observed in animals	Horng et al. (2017)
Microglia/Astrocytes	Mouse primary microglia and astrocytes	Experiments investigating the effect of LPS-induced mimic inflammatory conditions	Mutemberezi et al. (2018); Dambach et al. (2014)
Neurons/SIM-A9	Primary cortical and cerebellar neurons/Immortalized mouse microglia cells	Evaluation the role of microglia in neurodegeneration	Zhang et al. (2018a)
N11/N2A/MVEC(B3)	Murine microglial cells (N11)/Mouse neuroblastoma Nuro2A cell lines/Brain microvascular endothelial MVEC(B3) cells	An experimentally flexible tri-culture neuroinflammation model stimulated with LPS	Zheng et al. (2021)
3D BBB microvascular network	iPSC-endothelial cells/iPSC-brain pericytes/iPSC- astrocytes	Via vasculogenesis to accurately replicate the *in vivo* neurovascular organization	Campisi et al. (2018); Park et al. (2018); (Bergmann et al. (2018); Nzou et al. (2020)
	Neuron (Neu)/astrocyte (AC)/microglia (MG) differentiation from human neural progenitor cells (NPCs)	Study of human microglia recruitment, neuroinflammatory response and neuron/astrocyte damages	
	Multicellular 3D neurovascular unit organoid containing human brain microvascular endothelial cells, pericytes, astrocytes, microglia, oligodendrocytes and neurons	Model the effects of hypoxia and neuroinflammation on BBB function	
3D neuro-spheroids	Composition (e.g., neurons, astrocytes, and microglia), and environmental components (e.g., amyloid-β aggregates)	Construction of 3D cell culture and microenvironments	Simão et al. (2018); Cai et al. (2020)
